# Clinically Significant Lab Errors due to Vitamin B7 (Biotin) Supplementation: A Case Report Following a Recent FDA Warning

**DOI:** 10.7759/cureus.5470

**Published:** 2019-08-23

**Authors:** Ilana Rosner, Everett Rogers, Amanda Maddrey, David M Goldberg

**Affiliations:** 1 Internal Medicine, Nova Southeastern University's Dr. Kiran C. Patel College of Osteopathic Medicine, Davie, USA; 2 Biological Sciences, University of South Florida, St. Petersburg, USA; 3 Internal Medicine & Infectious Disease, Columbia University Vagelos College of Physicians and Surgeons, New York, USA

**Keywords:** biotin-streptavidin immunoassay, biotin, vitamin b7, lab errors, endocrinology

## Abstract

A 67-year-old female with a past medical history of multiple endocrine issues presented for follow-up subsequent to abnormal routine blood testing results. These included low thyroid stimulating hormone (TSH), low parathyroid hormone (PTH), and mildly elevated calcium levels. The presence of hypercalcemia and accompanying low PTH raised the concern for malignancy, while the depressed TSH indicated hyperthyroidism. Review of the patient’s medications revealed daily supplementation with 5 mg of vitamin B7 (biotin). The biotin was discontinued after suspecting the supplement was interfering with the patient's lab values. The labs were repeated one month later. The results showed normalized TSH, PTH, and calcium levels. The increasingly wide-spread use of biotin supplementation and the use of biotin as a component in many of the most common clinical assays has led to a trend of lab errors due to biotin interference. While some physicians are aware of the possibility of skewed results, steps need to be taken to prevent misdiagnosis. This includes ensuring that information about this issue is more widely disseminated, accurately accounting for a patient’s supplement use, reconciling proper clinical correlation with lab results, and promptly reporting when biotin is determined to be the cause of otherwise unexplained lab errors.

## Introduction

Biotin-associated interference is being increasingly recognized as a cause of abnormal lab results. Biotin is widely marketed and used for the promotion of hair, skin, and nail growth. Biotin-streptavidin binding kinetics make it ideal for use in a multitude of molecular tests and immunoassays. Depending on the specific design of the assay, high plasma concentrations of biotin can lead to falsely decreased or increased results of the molecule in question [1]. There have been recent reports published indicating instances of interference with assays for free thyroxine (T4), total T4, free triiodothyronine (T3), total T3, thyroid stimulating hormone (TSH), parathyroid hormone (PTH), testosterone, estradiol, 𝛽-human chorionic gonadotropin (β-hCG), ferritin, troponin, and various cancer markers [2-4].

While the Food and Drug Administration (FDA) has released a safety statement concerning the possibility of biotin interference, many patients may still be unknowingly consuming high enough levels to cause an interaction with common, but critically important lab tests [5,6]. Despite the relatively recent attention being paid to these interactions, many physicians may still be unaware of the potential for biotin interference resulting in incorrect diagnoses and treatment, unnecessary work-ups, and significant emotional stress to the patient as the result of false positive or negative lab work.

## Case presentation

A 67-year-old female with a past medical history of syndrome of inappropriate antidiuretic hormone (SIADH) and primary hyperparathyroidism status post partial parathyroidectomy presented for follow-up subsequent to abnormal routine blood testing results. These included low TSH, low PTH, and mildly elevated calcium levels. The presence of hypercalcemia (10.6 mg/dL) and accompanying low PTH (4.3 pg/mL) raised the concern for malignancy, while the depressed TSH (.24 mIU/L) indicated hyperthyroidism. Due to her previous endocrine issues, the patient was sent for further evaluation by endocrinology. Review of the patient’s medications revealed she was taking calcium, fenofibrate, sertraline, vitamin D3, ranitidine, as well as daily supplementation with 5 mg of biotin. Upon this discovery, the biotin was discontinued while the other medications were taken as prescribed. Her labs were repeated one month later, including a 24-hour urine calcium and creatinine level. These results showed a normalized TSH (3.3 mIU/L), PTH (21 pg/mL), and calcium (10.0 mg/dL), as shown in Table 1.

**Table 1:** Original lab values on 5 mg of biotin supplementation, and follow-up labs one month after discontinuation of biotin. PTH: Parathyroid hormone; TSH: Thyroid stimulating hormone.

Labs (Reference Range)	At Presentation	One Month Follow-up
PTH (15-65 pg/mL)	4.3 pg/ml	21 pg/ml
TSH (.465-4.683 mIU/L)	0.24 mIU/ml	3.30 mIU/ml
Calcium (6.8-10.5 mg/dL)	10.6 mg/dL	10 mg/dL
Calcium 24-hour urine (42-353 mg/24 hours)	N/A	250 mg/24 hours
Ionized Calcium (4.57-5.43 mg/dL)	N/A	5.25 mg/dL

## Discussion

Biotin is a water-soluble B vitamin naturally occurring in many foods, especially dairy products. It is required for various carboxylation reactions, acting as a co-enzyme in gluconeogenesis, lipogenesis, and fatty-acid synthesis [7]. It is widely marketed for its promotional effects on hair, skin, and nail growth, and is commonly sold as part of a multivitamin pill, or as a stand-alone supplement.

The current recommended dose of biotin for adults is 30 ug/day according to the Office of Dietary Supplements at the National Institute of Health, and the Food and Nutrition Board at the National Academy of Sciences, Engineering, and Medicine [7,8]. However, as seen with this patient, it is commonly found in supplements at levels from 5 mg up to 20 mg; a several hundred-fold increase [4-5]. While the amount of biotin naturally received through dietary means is not enough to interfere with clinical tests, the levels found in these supplements can generate significant errors.

A wide variety of laboratory tests rely on biotin as a component of the assay. These include endocrine and autoimmune tests, as well as those for malignancy and heart damage markers. In 2017, Holmes et al. reviewed the manufacturer's instructions for 374 methods performed by eight of the most popular immunoassay analyzers in the United States and found that 59% relied on biotin-based methods [4].

Biotin itself was initially discovered in 1927, but its role as a vitamin was not firmly established till several decades later [9]. Streptavidin is a glycoprotein whose name is derived from the bacteria in which it is found, *Streptomyces avidinii*, and the protein avidin, which is found in egg-whites. Streptavidin and avidin share an extremely powerful and specific ligand binding affinity for biotin [10]. Biotin is small and thus able to bind to a wide variety of molecules without altering their chemical properties. This property of biotin, along with streptavidin’s powerful and specific binding capabilities, makes it an excellent target for capture in laboratory assays [11].

**Figure 1:**
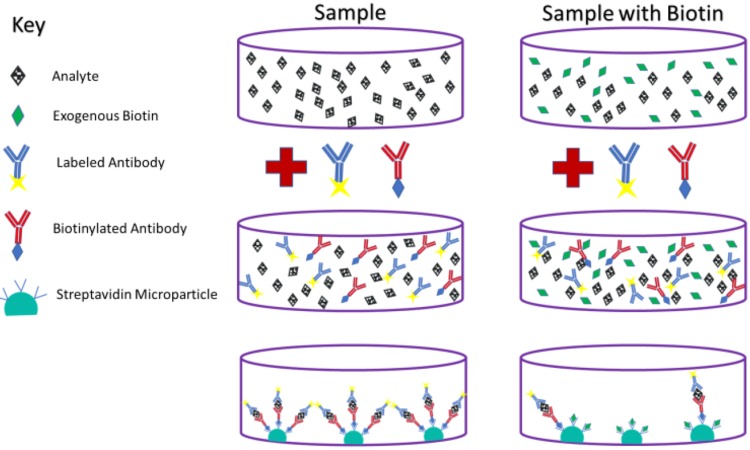
Sandwich assay with and without biotin. High concentrations of plasma biotin can lead to falsely decreased lab results in this type of assay.

Biotin mainly interferes with two types of clinical assays. The first is called a sandwich assay. Although the design of these assays as used by some companies makes them relatively immune to biotin interference, many are still susceptible. The sandwich assay is commonly used to determine levels of TSH, β-hCG, PTH, insulin, ferritin, pro b-type natriuretic peptide, prostate-specific antigen, and others [11]. These assays work by using two antibodies, both of which bind to the substance of interest, or in clinical chemistry terms, the analyte. The Fc region of one antibody is attached to the assay plate, while biotin is attached to the Fc portion of the other antibody in a process called biotinylation. Beads coated with streptavidin then bind the biotinylated antibodies, which can be used to create a fluorescent signal for detection [11]. When biotin is present in high concentrations from a sample obtained from a patient, it can saturate enough streptavidin on these beads to significantly limit their ability to bind the biotinylated antibodies. In this manner, high plasma levels of biotin in blood samples can lead to falsely lower analyte levels in assays that use a sandwich mechanism [4,11]. The mechanism of biotin interference in this type of assay can be seen in Figure 1.

**Figure 2:**
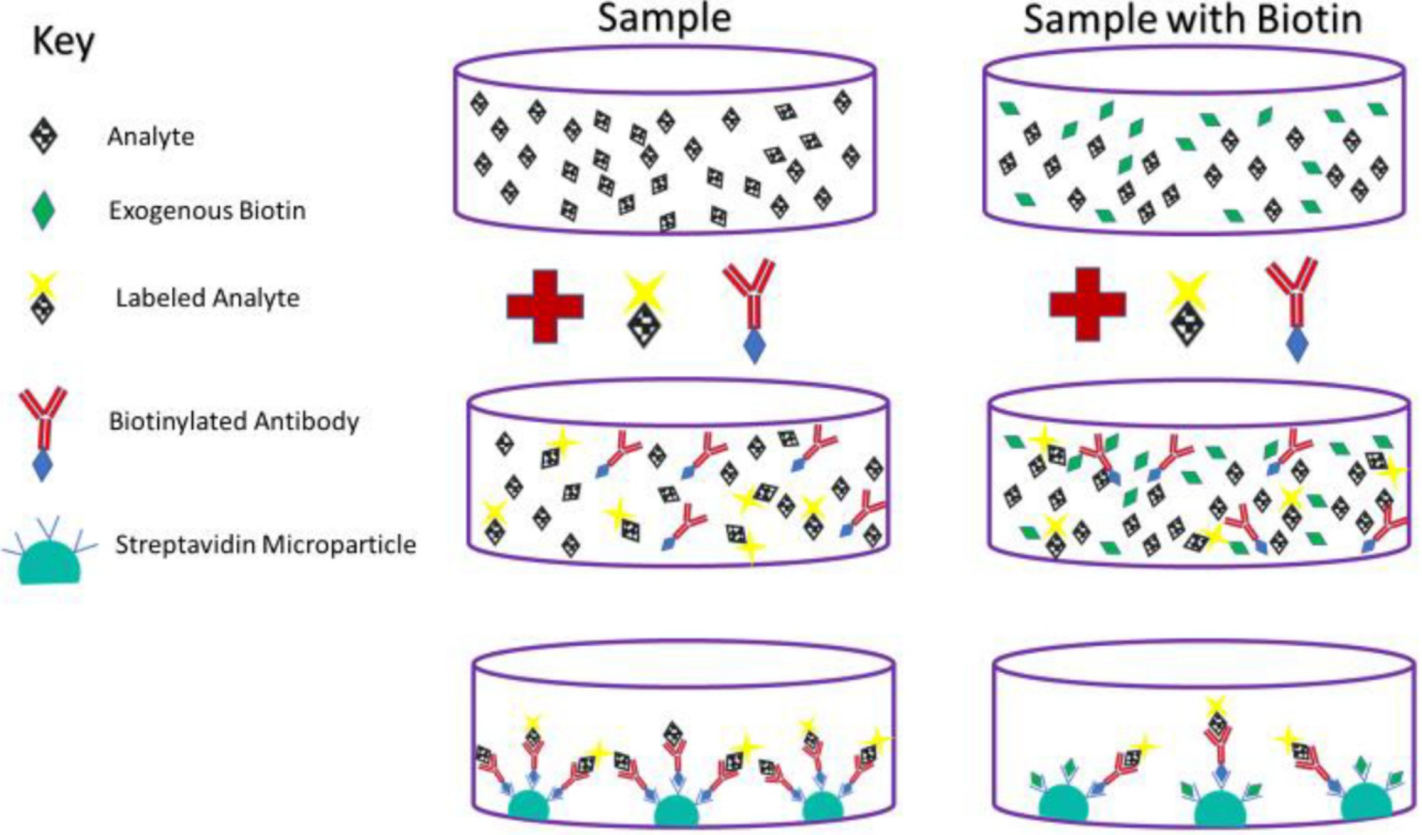
Competitive assay with and without biotin. High concentrations of plasma biotin can lead to falsely elevated lab results in this type of assay.

The other type of assay in which biotin interference can play a significant role is called a competitive assay. This type of assay is typically used to measure levels of small steroids and other molecules, including free T4, total T4, free T3, total T3, cortisol, and 25-hydroxyvitamin D (calcifediol) [11]. In a competitive assay, biotinylated antibodies bind the analyte. The biotinylated antibody then binds streptavidin on the surface of the plate. However, the analyte must compete with another molecule in the assay which is structurally similar to the analyte, but contains a label that can be detected [11]. If the analyte is present in high concentration, fewer antibodies will be bound to the labeled competitor, resulting in a weaker signal. Conversely, if the analyte is present in low concentrations, the antibodies will bind more labeled competitors, and the signal will be stronger. Therefore, if biotin is highly concentrated in the patient sample, it will bind more streptavidin on plate, leading to less binding of antibody (regardless of attachment to analyte or labeled competitor), a weaker signal, and falsely elevated results [4,11]. The mechanism of biotin interference in this type of assay can be seen in Figure 2.

Concurrent to its increased popularity as a supplement, biotin is also increasingly being used as an alternative treatment for certain diseases. When administered at doses of 300 mg/day, one clinical trial showed clinical improvement in 13 out of 103 patients with multiple sclerosis, versus none in the placebo control arm [12]. More modest levels of biotin supplementation have been shown to significantly reduce fasting glucose levels in patients with type 2 diabetes mellitus, as well as triglyceride levels in those with and without type 2 diabetes mellitus [13-15]. However, some of the results from these data are conflicting.

Biotin also plays an important role in the treatment of several inherited metabolic disorders. In fact, one of the earlier reports warning about biotin interference in lab tests came from a 2016 report by Kummer et al. [3]. In this report, the authors describe the development of lab results indistinguishable from Graves' disease (i.e., excessively elevated free T3 and T4, low thyrotropin, and elevated levels of anti-thyrotropin receptor antibodies) in six children receiving high dose biotin in the setting of inherited metabolic disorders. Due to biotin’s under-recognition as a potential confounder of lab tests, three of these children received methimazole for treatment of their apparent hyperthyroidism (two of them for over a year) before the culprit was found [3]. This report highlights the difficulties in distinguishing abnormal lab results from biotin-induced errors, and the very high level of clinical suspicion needed in order to avoid potentially harmful intervention.

Many patients may be unaware of the presence of biotin in their multivitamins or prenatal vitamins, and as a result, it may go unreported to their primary care providers [1]. It is important to note that high levels of biotin supplementation strictly interfere with *in vitro* lab tests. At the supplement levels being discussed, it would not generally cause toxicity and would not lead to disruption of endocrine, metabolic, or other pathways. In other words, the patient’s abnormal blood results would not be reflected in the patient, who is likely to be asymptomatic. It is therefore important to maintain a high index of suspicion and ask about biotin supplementation levels when an otherwise asymptomatic patient presents with abnormal results. When clinical suspicion of biotin interference is high, it has been suggested that a patient waits at least several days before repeating the abnormal tests [1,5,11]. Given the relatively recent attention being paid to these interactions, many physicians may still be unaware of the potential for biotin interference, resulting in incorrect diagnoses and treatment, unnecessary work-ups, and significant emotional stress, as seen with this patient.

The 2017 safety report released by the FDA has alerted the public and health care workers about the possibility of biotin interference, but many patients may still be unknowingly consuming high enough levels to cause an interaction with common, but critically important lab tests [5-6]. The FDA currently recommends that health care providers consider biotin interference as a possible source of error if a lab test result does not match a patient’s clinical presentation, and that if it is determined that biotin is the cause of the error, that this be reported to the lab test manufacturer, as well as the FDA through MedWatch, the FDA Safety Information and Adverse Event Reporting program [6].

## Conclusions

Biotin supplementation remains an under-recognized cause of abnormal lab results. It has been shown to skew a wide variety of laboratory tests including troponin, thyroid, parathyroid, and electrolyte assays, as well as many others. These lab errors can cause emotional strain on the patient, lead to costly and unnecessary work-ups, and potentially harmful and unnecessary interventions. A high degree of suspicion is required on the part of the clinician in order to catch and correctly attribute these lab errors to biotin over-supplementation. This requires not only that healthcare providers be knowledgeable about this interaction, but also that they can account for all of the ingredients in reported supplements. When biotin interference is identified as the source of error, this information should be reported to the lab test manufacturer and the FDA.
